# Establishing a library of resources to help people understand key concepts in assessing treatment claims—The “Critical thinking and Appraisal Resource Library” (CARL)

**DOI:** 10.1371/journal.pone.0178666

**Published:** 2017-07-24

**Authors:** John C. Castle, Iain Chalmers, Patricia Atkinson, Douglas Badenoch, Andrew D. Oxman, Astrid Austvoll-Dahlgren, Lena Nordheim, L. Kendall Krause, Lisa M. Schwartz, Steven Woloshin, Amanda Burls, Paola Mosconi, Tammy Hoffmann, Leila Cusack, Loai Albarqouni, Paul Glasziou

**Affiliations:** 1 James Lind Initiative, Oxford, United Kingdom; 2 Minervation Ltd., Oxford, United Kingdom; 3 Global Health Unit, Institute of Public Health, Oslo, Norway; 4 Høgskulen på Vestlandet, Centre for Evidence-Based practice, Bergen, Norway; 5 Global Development Program, Bill & Melinda Gates Foundation, Seattle, Washington, United States of America; 6 Medicine in the Media Program, The Dartmouth Institute, Hanover, New Hampshire, United States of America; 7 School of Health Sciences, City University London, London, United Kingdom; 8 Laboratorio di ricerca sul coinvolgimento dei cittadini in sanità, Istituto di Ricerche Farmacologiche Mario Negri, Milano, Italy; 9 Centre for Research in Evidence-Based Practice, Faculty of Health Sciences and Medicine Bond University, Queensland, Australia; Public Library of Science, FRANCE

## Abstract

**Background:**

People are frequently confronted with untrustworthy claims about the effects of treatments. Uncritical acceptance of these claims can lead to poor, and sometimes dangerous, treatment decisions, and wasted time and money. Resources to help people learn to think critically about treatment claims are scarce, and they are widely scattered. Furthermore, very few learning-resources have been assessed to see if they improve knowledge and behavior.

**Objectives:**

Our objectives were to develop the Critical thinking and Appraisal Resource Library (CARL). This library was to be in the form of a database containing learning resources for those who are responsible for encouraging critical thinking about treatment claims, and was to be made available online. We wished to include resources for groups we identified as ‘intermediaries’ of knowledge, i.e. teachers of schoolchildren, undergraduates and graduates, for example those teaching evidence-based medicine, or those communicating treatment claims to the public. In selecting resources, we wished to draw particular attention to those resources that had been formally evaluated, for example, by the creators of the resource or independent research groups.

**Methods:**

CARL was populated with learning-resources identified from a variety of sources—two previously developed but unmaintained inventories; systematic reviews of learning-interventions; online and database searches; and recommendations by members of the project group and its advisors. The learning-resources in CARL were organised by ‘Key Concepts’ needed to judge the trustworthiness of treatment claims, and were made available online by the James Lind Initiative in Testing Treatments interactive (TTi) English (www.testingtreatments.org/category/learning-resources).TTi English also incorporated the database of Key Concepts and the Claim Evaluation Tools developed through the Informed Healthcare Choices (IHC) project (informedhealthchoices.org).

**Results:**

We have created a database of resources called CARL, which currently contains over 500 open-access learning-resources in a variety of formats: text, audio, video, webpages, cartoons, and lesson materials. These are aimed primarily at ‘Intermediaries’, that is, ‘teachers’, ‘communicators’, ‘advisors’, ‘researchers’, as well as for independent ‘learners’. The resources included in CARL are currently accessible at www.testingtreatments.org/category/learning-resources

**Conclusions:**

We hope that ready access to CARL will help to promote the critical thinking about treatment claims, needed to help improve healthcare choices.

## Introduction

People are confronted every day by claims about the effects of treatments. Many, if not most, of these claims are unsupported by evidence [1][2], meaning they can put patients at risk of harm[3]. Where claims seek to sell a treatment, patients may spend their own money on treatments of no known benefit, or may seek inappropriate treatments, which can waste public resources on a large scale [4]. Additionally, misleading claims can exacerbate peoples’ natural tendency to overestimate the benefits of treatments and to underestimate their potential risks[5]. This might lead patients to seek inadequately evaluated treatments with unrecognised adverse effects[6][7]or avoid treatments likely to help them [8].

Similarly, health professionals may treat patients using methods that have been insufficiently evaluated [9] or act based on comparisons which have not assessed treatments using patient-valued outcomes. Professionals may also exploit treatment claims to fulfil their own, conflicted, interests [10]. Finally, as treatment claims often contradict each other and cause confusion, patients may simply become disillusioned with, and lose respect for, the relevance of research evidence [11].

To address these problems, people need to be able to think critically about the treatment claims they come across [12]. They should be able to assess the strength of the supporting evidence underlying a treatment claim, the conflicts of interest among those making the claims, and the relevance of the research evidence to their healthcare needs [13][14]. These processes are part of a wider range of skills people should use to make well-reasoned decisions on a day-to-day basis.

Increased general knowledge about how to evaluate treatment claims would mean people could more confidently use information about health claims to promote their own health [15][16][17][18][19][20]. It would mean that health professionals, together with their patients, could make more balanced decisions [21]. It would also enable greater patient involvement in shared-decision making [22][23], with fewer resources wasted and fewer avoidable harms.

Despite its importance, the promotion of critical thinking—the use of logic and evidence to assess the strength of the arguments that underlie claims—is not widespread. Furthermore, the effects of learning-resources that aim to do this have very rarely been formally evaluated. This lack of assessed learning-resources disadvantages people who wish to think for themselves about treatment decisions. To address this, it would be valuable to collate and make accessible an inventory of relevant, and where possible formally evaluated, existing learning-resources to help people who are responsible for teaching others how to assess treatment claims.

**Fig 1:**
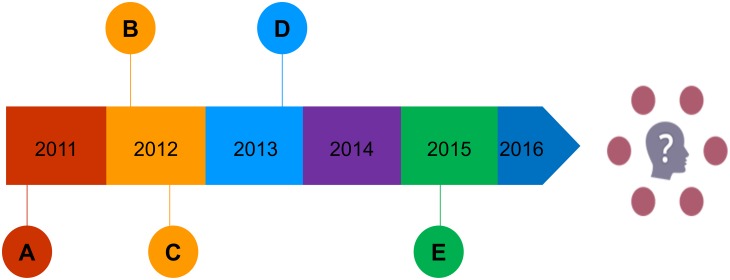
Timeline demonstrating the stages that lead to the development of CARL. (A) Inventory by L Kendall Krause created. (B) The James Lind Initiative begins adding resources to Testing Treatments interactive. (C) European Communication on Research Awareness Needs (ECRAN) inventory created. (D) The Informed Healthcare Choices Project (IHC) formed, Members begin to identify useful resources. (E) Discussions between IHC and JLI lead to expressions of support for a new library, JLI agrees to coordinate development until 2019.

There have been efforts since 2011 to create an inventory of learning-resources for this purpose [24][25] (Fig 1). In October 2015, in Vienna, the Informed Healthcare Choices (IHC) project group and the James Lind Initiative (JLI) co-convened an international, multidisciplinary workshop for people interested in helping others to make sense of treatment claims. One of the presentations at the workshop was used to gauge support for a further attempt to develop and maintain an inventory of learning-resources, as well as to inform the development and evaluation of new resources. After written expressions of interest were submitted, it was agreed that the JLI, which is funded by the National Institute for Health Research (NIHR), would oversee the formation of such an inventory. Thus, we have created the Critical thinking and Appraisal Resource Library (CARL).

In summary, our objectives were to develop the Critical thinking and Appraisal Resource Library for those who are responsible for encouraging critical thinking about treatment claims. These include: teachers of schoolchildren, undergraduates and graduates, for example those teaching evidence-based medicine, or those communicating treatment claims to the public. In selecting resources, we draw attention to those resources that have been formally evaluated with pre-defined criteria, for instance by the resource creator or by independent research groups.

## Methods

### Populating CARL

Candidate learning-resources for possible inclusion in CARL have been identified in previously compiled inventories [25][26] and through:

1. the Informed Health Choices project [www.informedhealthchoices.org]
2. *Testing* Treatments [3] and ‘Testing Treatments *interactive*’ [www.testingtreatments.org]
3. the *James Lind Library* [27] [www.jameslindlibrary.org]
4. systematic reviews of educational interventions by Nordheim [26], Austvoll-Dahlgren[28]; Cusack [29]; and Albarqouni (in preparation).
5. the Educational Endowment Foundation [https://educationendowmentfoundation.org.uk]
6. the Times Education Service. [https://www.tes.com/teaching-resources]
7. Online searches of databases such as ERIC, PubMed, and in search engines for resources related to keywords.

When resources were identified, we made attempts to discover if the resource had been formally evaluated to see if their intended learning objectives had been achieved. Where formal evaluations were found, these were assessed for eligibility by pre-defined criteria [see S3 Appendix].

### Development of CARL

#### Key concepts for organising resources in CARL

CARL is a database of learning resources organized around a list of Key Concepts that people need to understand to assess the trustworthiness of treatment claims, as developed by the IHC project[30] (www.testingtreatments.org/key-concepts-for-assessing-claims-about-treatment-effects) [see S1 Appendix]. Our definition of ‘treatments’ includes any action intended to improve health or relieve suffering. These include: changes in behaviour, screening programmes, drugs, surgery, physical and psychological treatments; and public health and healthcare system changes.

The IHC Key Concepts have been largely derived from the content of *Testing Treatments* [3], a book written for the public (currently in over a dozen languages; www.testingtreatments.org), as well as from a variety of other resources with the same goal. Researchers and learners in Norway, the UK, Uganda and Australia identified a list of over 30 Key Concepts, the process of which has been described by Austvoll-Dahlgren et al. in 2015 [30].

We organised these concepts under three headings (see S1 Appendix):

Claims: are they justified? currently 12 Key Concepts
Comparisons: are they fair and reliable? currently 17 Key Concepts
Choices: are the findings relevant to you? currently 5 Key Concepts

As additional key concepts are identified and agreed, they will be added with explanations and illustrations.

#### Including and excluding resources

The principal eligibility assessor (JCC) judged whether candidate resources should be included based on their relevance to the IHC Key Concepts. For learning-resources of questionable eligibility, IC and other members of the CARL editorial group also assessed the resources for inclusion. Resources to teach content specific to clinical information, such as decision aids, have not been included. Given the varied formats of resources, resource format-specific inclusion criteria were also agreed (see S2 Appendix).

Learning-resources that have been formally evaluated, for instance in randomised trials, are of particular importance. As previously mentioned, separate inclusion criteria were agreed for assessing the suitability of formal evaluations (S3 Appendix).

#### Coding resources

To facilitate navigation of CARL, each resource has been tagged with categories deemed important by the editorial group:

1. Unique resource identification code.
2. Name/Title (as stated by the resource host)
3. Format: Text; Audio; Video; Cartoons; Websites/pages; and Lessons (including presentations, e-Learning modules and specific materials for teaching students, such as learning exercises or worksheets)
4. Reference/URL
5. Language
6. IHC Key Concepts to which the resource is relevant
7. Effects of a resource on knowledge/understanding, with links to reports of the evaluations. Where reports are publications with restricted access, the JLI has summarised the findings on the website www.testingtreatments.org.
8. Target user groups commonly mentioned by resource developers, categorized further for CARL, as:
  - Teachers, including teachers of primary school, secondary school (coded as ‘School Teachers’), and teachers of undergraduate and postgraduate health profession students (coded as ‘Higher Level Teachers’).
  - Communicators, such as journalists and science writers.
  - Advisors, for example, those who wish to help improve decision-making by policy makers, or by members of research ethics committees.
  - Researchers who wish to assess the effects of learning-resources designed to teach Key Concepts.

Learners, anyone who wishes to teach themselves how to assess treatment claims, for example, interested members of the public (coded as General Learners), and undergraduate, postgraduate or professional students (coded as Higher Level learners).

### Providing access to the resources in CARL

TTi (www.testingtreatments.org) is the first website providing access to the learning-resources in the CARL database and we hope that the library may be used by other websites in the future. The website has been redesigned to use the Key Concepts as the framework for organising its content, specifically into three groups: ‘Claims: are they justified?’; ‘Comparisons: are they fair and reliable?’; and ‘Choices: are the findings relevant?’.

Resources are accessed from the navigation menu, and content can be filtered according to specific interests, such as Target Users, and Resource Format. Short descriptions of each resource have also been added. Formally evaluated resources are listed higher in search results and are clearly demarcated with a bold, green tick. In addition, an informal review process will be implemented: users will be able to ‘like’ or ‘recommend’ resources, and those with the highest number of likes will be ranked higher in the search results accordingly.

We have instituted an ongoing process to evaluate the website, including experimental testing of functionality, presentation and content. A software platform has also been installed to support randomised comparisons of alternative ways of promoting learning about the IHC Key Concepts. The process and results of these evaluations will be described and submitted for publication.

## Results

The website ‘Testing Treatments *interactive*’ currently provides access to over 300 open-access resources in CARL and more are being added following the same search and screening processes described earlier. We expect this number to grow with periodic further searches and through CARL users proposing additional resources using an online suggestion form.

The IHC Key Concepts that currently have the highest average number of resources are those grouped under ‘*Comparisons*: *are they fair and reliable*?*’*. This contains IHC Key Concepts relevant to randomised trials and systematic reviews, amongst other topics. The Key Concept group with the lowest average number of resources per Key Concept is *‘Choices*: *are the findings relevant*?*’*, which contains IHC Key Concepts that address how users may apply evidence and treatment claims in their own decisions. The average number of resources per Key Concept may change as further searches are conducted, but it may also highlight which areas are lacking, and therefore inform development of additional resources by interested parties.

**Fig 2:**
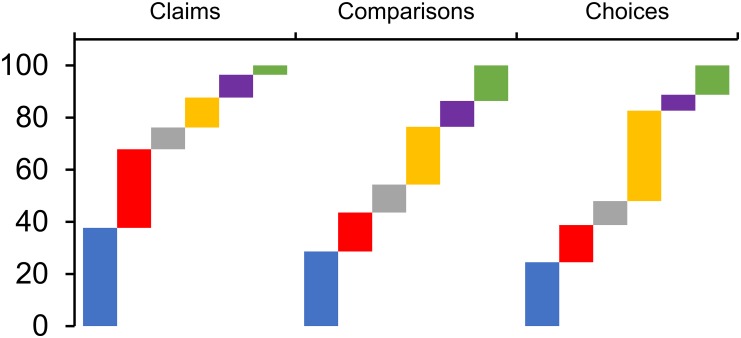
Graph to show the proportion of resources that are of each format in the CARL database. Vertical axis is percentage of total resources of the specified target users. Horizontal represents the Key Concept group in question. (Blue) Text. (Red) Audio. (Grey) Videos. (Yellow) Websites. (Purple) Cartoons. (Green) Lessons.

Of the resources that have been formally evaluated [31][32][33][34][35][36][37][38][39][40][41][42][43][45][46] the Key Concept group to which most are coded is ‘*Comparisons*: *are the results fair and reliable*?’ and this reflects that many of the resources currently available are used for teaching evidence-based medicine. The group that has the fewest formally evaluated resources is ‘*Choices*: *are the findings relevant*?*’*. This is likely to be because there are fewer Key Concepts in this group. Work is ongoing to expand the number of Key Concepts in this section. The most common formats of resources are Text and Audio, mostly derived from *Testing* Treatments [3]. The least common format of resources is currently Lessons. (Fig 2).

Specific IHC Key Concepts with relatively few resources include:

- Increasing the amount of a treatment does not necessarily increase the benefits of a treatment and may cause harm
- Beliefs about how treatments work are not reliable predictors of the actual effects of treatments
- Treatment decisions should take account of both beneficial and harmful effects
- Average differences between treatments can be misleading
- The treatments evaluated in fair comparisons may not be relevant or applicable
- Results for a selected group of people within fair comparisons can be misleading

Additionally, relatively few resources for teachers of children and young people have been identified, as well as few for ‘advisors’, ‘communicators’ and ‘researchers’.

**Fig 3:**
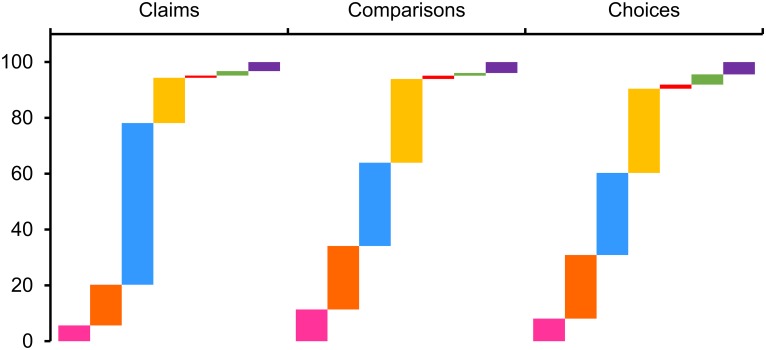
Graph to show the proportion of resources that are targeted at each Target User group in the CARL database by each of the Key Concept groups. Vertical axis is percentage of resources in a group. Horizontal is the Key Concept group in question. (Pink) Teachers of school age children. (Orange) Teachers of students in further education. (Light blue) Independent learners with general interests. (Yellow) Higher level learners, such as those in further education or with higher level knowledge. (Red) Advisors, e.g. policymakers. (Green) Communicators, e.g. journalists. (Purple) Researchers interested in developing educational resources.

The largest number of learning-resources is for ‘Learners’, divided into: ‘General Learners’ i.e. those who wish to educate themselves about the Key Concepts, and ‘Higher Learners’, including undergraduate and postgraduate health profession students (see Fig 3). Many of the learning-resources included in CARL had no specified Target User Group when originally identified in searches. These have been coded to specific user groups after discussion. Mostly, where resource target user groups were not specified, the resources were coded as for ‘General Learners’. This is because they may be relevant to one or more of the ‘Intermediaries’ listed above, or to anyone who wants to teach themselves how to use Key Concepts to assess the validity of treatment claims.

## Discussion

The Critical thinking and Appraisal Resource Library—CARL—has been created to help those who teach people how to evaluate claims about the effects of treatments. This report describes how CARL has been developed. We hope CARL will become an increasingly improved and indispensable directory for teachers, communicators, advisors, researchers and learners.

Systematic and online searches for additional learning-resources relating to each Key Concept will be conducted periodically. It is anticipated that additional resources will be recommended to us by users of the site and by those who share our interests.

With the initial phase of resource identification and coding complete, future emphasis will be on encouraging formal evaluation of the effects of resources on understanding and ability to apply Key Concepts, by independent groups who express interest in the project.

Some examples of formally evaluated resources include the booklet ‘Know Your Chances’ [42], which was compared objectively with a decision aid in two randomised controlled trials, in populations of different socioeconomic backgrounds [34]. Both trials found that people who had read ‘*Know Your Chances’* were better able to understand risk than those who had used the decision aid.

Another formally evaluated resource is ‘Thinking, Doing, Talking Science’ [41], which promotes an alternative method of teaching science to primary school children, with an emphasis on greater cognitive challenge, interactivity and scientific reasoning [40]. This approach was evaluated in a randomised controlled trial that demonstrated an average increase in pupil progress in science, compared with control children, equivalent to two extra terms of teaching. It also showed that progress was relatively greater in students from poorer socioeconomic backgrounds. This study did not measure pupils’ understanding or ability to apply Key Concepts, but rather how well they did in national exams. It demonstrated that, in these terms, scientific reasoning improved.

A challenge in formally evaluating the impact of learning-resources is how to define and measure their effects. The IHC Project has created the Claim Evaluation Tools (Austvoll-Dahlgren et al. Submitted), a collection of multiple choice questions that test knowledge of the Key Concepts (www.testingtreatments.org/create-test-claim-evaluation-tools-database). These have been validated in Ugandan primary school children [44] for which information is publicly available through www.testingtreatments.org, and will potentially help interested researchers to conduct formal evaluations of resources.

In a randomised trial involving 120 schools, the Claim Evaluation Tools have been used to assess the effects of IHC primary school learning-resources (including a comic book text) on primary school children in Uganda [45]. In a linked trial, IHC resources were. used to evaluate the effects on parents of primary school children who have listened to an IHC podcast [46].

Helping children to think critically about treatment claims is important, particularly at an age when they may be more open to thinking critically, and have the time to learn the skills needed. Acquiring these skills as young children could provide a foundation upon which to strengthen their ability to make reasoned decisions. Indeed, the Key Concepts upon which the content of CARL is organised apply not only to claims about the effects of treatments, but also to other arguments and assertions—economic, social and political.

To summarise, the James Lind Initiative has coordinated the development of an open-access Critical thinking and Appraisal Resource Library (CARL), to help teachers and learners to increase general knowledge of Key Concepts relevant to assessing claims about the effects of treatment. These resources are currently available at www.testingtreatments.org.

## The Fair Comparisons Network

Expressions of interest in CARL have been encouraging and have led to the development of a ‘Fair Comparisons Network’. This an informal list of individuals who share a common interest in promoting critical thinking and critical appraisal skills. The network is open for other members with similar interests to join and to help identify resources for inclusion in CARL. We hope that, as the Fair Comparisons Network expands, people who share an interest in a specific target user group or resource format will work together to increase the proportion of that are formally evaluated learning-resources. People who are not already members of the Fair Comparisons Network can join it by emailing Patricia Atkinson at patkinson@jameslind.net. Emails should include Name, Contact Information, and areas of interest.

## Supporting information

S1 Appendix The key concepts. (DOCX)

S2 Appendix Selection criteria for learning-resources. (DOCX)

S3 Appendix Selection criteria for evaluations of learning-resources. (DOCX)
