# Supplementary material for: Establishing a library of resources to help people understand key concepts in assessing treatment claims—The “Critical thinking and Appraisal Resource Library” (CARL)
Source: PLoS One. 2017 Jul 24;12(7):e0178666. doi: 10.1371/journal.pone.0178666 (PMC5524286; doi:10.1371/journal.pone.0178666)
Supplement: S1 Appendix — (DOCX) [file pone.0178666.s001.docx]

**1. Claims: are they justified?**

1-1 Treatments may be harmful

1-2 Personal experiences or anecdotes (stories) are an unreliable basis for assessing the effects of most treatments

1-3 A treatment outcome may be associated with a treatment, but not caused by the treatment

1-4 Widely used treatments or treatments that have been used for a long time are not necessarily beneﬁcial or safe

1-5 New, brand-named, or more expensive treatments may not be better than available alternatives

1-6 Opinions of experts or authorities do not alone provide a reliable basis for deciding on the beneﬁts and harms of treatments

1-7 Conﬂicting interests may result in misleading claims about the effects of treatments

1-8 Increasing the amount of a treatment does not necessarily increase the beneﬁts of a treatment and may cause harm

1-9 Earlier detection of disease is not necessarily better

1-10 Hope or fear can lead to unrealistic expectations about the effects of treatments

1-11 Beliefs about how treatments work are not reliable predictors of the actual effects of treatments

1-12 Large, dramatic effects of treatments are rare

**2. Comparisons: are they fair and reliable?**

2-1 Evaluating the effects of treatments requires appropriate, fair comparisons

2-2 Apart from the treatments being compared, the comparison groups need to be similar, i.e. ’like needs to be compared with like’

2-3 People’s experiences should be counted and analysed in the group to which they were allocated

2-4 People in the groups being compared need to be cared for similarly (apart from the treatments being compared)

2-5 If possible, people should not know which of the treatments being compared they are receiving

2-6 Outcomes should be measured in the same way (fairly) in the treatment groups being compared

2-7 It is important to measure outcomes in everyone who was included in the treatment comparison groups

2-8 The results of single comparisons of treatments can be misleading, the results of all relevant fair comparisons should be considered.

2-09 Reviews of treatment comparisons that do not use systematic methods can be misleading

2-10 All fair comparisons and outcomes should be reported

2-11 Small studies in which few outcome events occur are usually not informative and the results may be misleading

2-12 Subgroup analysis may be misleading

2-13 Relative measures of effects can be misleading

2-14 Average measures of effects can be misleading

2-15 The use of p-values to indicate the probability of something having occurred by chance may be misleading; conﬁdence intervals are more informative

2-16 Saying that a difference is statistically signiﬁcant or that it is not statistically signiﬁcant can be misleading

2-17 Don’t confuse “no evidence of effect” with “evidence to suggest no effect”

**3. Choices: are the findings relevant?**

3-1 Do the outcomes measured matter to you?

3-2 Are you very different from the people studied?

3-3 Are the treatments practical in your setting?

3-4 How certain is the evidence?

3-5 Do the advantages outweigh the disadvantages?
