# Supplementary material for: Establishing a library of resources to help people understand key concepts in assessing treatment claims—The “Critical thinking and Appraisal Resource Library” (CARL)
Source: PLoS One. 2017 Jul 24;12(7):e0178666. doi: 10.1371/journal.pone.0178666 (PMC5524286; doi:10.1371/journal.pone.0178666)
Supplement: S2 Appendix — (DOCX) [file pone.0178666.s002.docx]

**Key Criteria**

*i) Must be a* ***learning-resource****; i.e. text, video, software, or other material that assists learners (anyone wanting to learn) to understand and apply one or more of the* ***Key Concepts***

*ii) Must be* ***freely available***

*iii) Must provide an explanation, and preferably an example* for one or more of the* ***Key Concepts***

**Text (includes books, book chapters and articles^†^)**

*Must satisfy i-iii*

- Individual chapters or sections in books are included only if the majority of the content in the rest of the book does not help the target audience to understand and apply one or more of the **Key Concepts**, but the chapter or section in question does.

**Video (also encompasses animations and interactive games)**

*Must satisfy i-iii*

- Where the main content of the video does not address a Key Concept but part of the video does, the section can only be used in isolation (with permission) if it does not require further explanation, i.e. is not dependent on context provided by the main content.

**Audio (includes songs, audiobooks, podcasts, recorded interviews and talks)**

*Must satisfy i-iii.*

- Where the main content of the audio does not address a Key Concept but part of the audio does, the section can only be used in isolation (with permission) if it does not require further explanation, i.e. is not dependent on context provided by the main content.

**Lessons (includes PowerPoint presentations, recorded presentations, worksheets, lesson plans, etc.)**

*Must satisfy i-iii*

- Lesson plans must provide sufficient detail to be replicable, eg, objectives, sequence, timings, materials, etc)
- PowerPoint presentations must be interpretable by people who are not methodologists i.e. must include notes, or be sufficiently clear so that notes are not needed.
- Quizzes (test yourself resources) must provide access to the correct answers and explanations. This does not apply to learning questions that are embedded in a learning-resource, such as those in workshop materials.
- Checklists must include instructions or explanations.
- Critical Appraisal Tools are only included if they explain the reasoning for steps in the critical appraisal process as relates to the Key Concepts.

**Cartoons**

*Must satisfy i-iii* *or address a term included in the GET-IT jargon buster.*

- Cartoons that are related to **Key Concepts** or **GET-IT** terms, but do not help people to understand the concept or term, are excluded.

**Websites**

*Must satisfy i-iii*

- Specific webpages and parts of websites are included in isolation only if the majority of content on the site does not help the target audience to understand and apply one or more of the **Key Concepts**, but the parts of the website in question do.
