# Supplementary material for: Establishing a library of resources to help people understand key concepts in assessing treatment claims—The “Critical thinking and Appraisal Resource Library” (CARL)
Source: PLoS One. 2017 Jul 24;12(7):e0178666. doi: 10.1371/journal.pone.0178666 (PMC5524286; doi:10.1371/journal.pone.0178666)
Supplement: S3 Appendix — (DOCX) [file pone.0178666.s003.docx]

**Key Criteria**

*iv) Must evaluate a learning-resource that meets the above criteria*

*v) Must include a description of the methods that were used*

**Evaluations of effects**

*Must satisfy iv and v*

- Must be a comparison. Comparisons of knowledge after an intervention with knowledge before are eligible, but ’after only’ evaluations (i.e. without a comparison group or pretest measure) will be excluded.
- Must have a clearly defined outcome measure; e.g. knowledge, skills, attitudes, behaviour.
- Measurement of knowledge must be objective.
- Must report the difference between the comparison groups quantitatively.

**Evaluations of user experiences or factors that facilitate or hinder use of a learning-resource, including pilot studies and user testing (formative evaluations)**

*Must satisfy iv and v*

- Must have implications for the design of the learning-resource.
- Can use qualitative or quantitative methods.

**Process evaluations**

*Must satisfy iv and v*

- Must be linked to an assessment of effects.
- Address one or more questions related to obtaining a better understanding of how or why a learning-resource works or does not work; e.g. fidelity (whether the resource was used as intended), explaining variation in effects, identifying potential unintended effects, identifying factors that might improve the resources or how they are used, or that might facilitate or hinder scaling up use of the resource.

**Reviews (e.g. book reviews)**

Are excluded.

**Evaluations of instructed educational interventions (syllabuses for educational courses; journal clubs)**

Are excluded unless detailed information of the interventions and teaching resources used are provided and explained, such that the intervention can be reliably repeated without requiring additional information.
